# A Comprehensive Pan-Cancer Analysis of the Tumorigenic Role of Matrix Metallopeptidase 7 (MMP7) Across Human Cancers

**DOI:** 10.3389/fonc.2022.916907

**Published:** 2022-06-17

**Authors:** Nana Meng, Yaguang Li, Pengcheng Jiang, Xuefeng Bu, Jifei Ding, Yan Wang, Xiaodong Zhou, Feng Yu, Yongjun Zhang, Jie Zhang, Leizhou Xia

**Affiliations:** ^1^Department of Ophthalmology, Affiliated People’s Hospital, Jiangsu University, Zhenjiang, China; ^2^Department of Ophthalmology, Zhenjiang Kangfu Eye Hospital, Zhenjiang, China; ^3^Department of Kidney Transplantation, Second Xiangya Hospital of Central South University, Changsha, China; ^4^Department of General Surgery, Affiliated People’s Hospital, Jiangsu University, Zhenjiang, China; ^5^Department of Thoracic Surgery, Jiangsu Province Hospital on Integration of Chinese and Western Medicine Affiliated to Nanjing University of Chinese Medicine, Nanjing, China; ^6^Department of Orthopedic, Affiliated People’s Hospital, Jiangsu University, Zhenjiang, China

**Keywords:** bioinformatics, MMP7, prognosis, cancer-associated fibroblasts, tumor-infiltrating immune cells, pan-cancer

## Abstract

Growing evidence has shown the oncogenic function of matrix metallopeptidase 7 (MMP7) in various tumors. However, no systemic pan-cancer analysis on the association between MMP7 and different cancers based on big clinical data is available. TIMER2, GEPIA2, UALCAN, cBioPortal, String, Metascape, and other web databases were searched in the present study. Generally, *MMP7* expression is significantly upregulated in most The Cancer Genome Atlas (TCGA) cancer types compared to the paired normal controls, yet is downregulated in tumor tissues of invasive breast carcinoma (BRCA), kidney chromophobe (KICH), kidney renal clear cell carcinoma (KIRC), liver hepatocellular carcinoma (LIHC), and skin cutaneous melanoma (SKCM). MMP7 protein expression is notably higher in the primary tumor tissues of colon cancer, lung adenocarcinoma (LUAD), and uterine corpus endometrial carcinoma (UCEC) than in normal tissues and is significantly lower in the primary tumor tissues of breast cancer, clear cell renal carcinoma, and ovarian cancer. Furthermore, *MMP7* expression is strongly associated with pathological stages, clinical outcomes, tumor mutational burden (TMB), and microsatellite instability (TSI). Gene amplification was detected in most TCGA cancer types. In addition, the missense mutation is the primary type of MMP7 genetic alteration in tumors. Significant positive correlations between *MMP7* expression and cancer-associated fibroblasts (CAFs) have been demonstrated in most TCGA cancers. *MMP7* expression was also found to be positively correlated with infiltration of dendritic cells and macrophages in some specific tumor types. Functional enrichment analysis by the Kyoto Encyclopedia of Genes and Genomes (KEGG) pathways and gene ontology (GO) methods revealed that RNA processing and DNA damage checkpoints might reveal the pathogenetic mechanisms of MMP7. This pan-cancer analysis provides a clear panorama for the tumorigenic roles of MMP7 across different cancer types. Moreover, MMP7 could be a potential drug therapeutic target in such cancers.

## Introduction

Matrix metalloproteinases (MMPs) are a family of structural-related zinc-dependent endopeptidases which can degrade almost all kinds of extracellular matrix proteins, including collagens and gelatins (1, 2), and can regulate the cleavage of cell surface receptors and also the release of apoptotic ligands and cell surface molecules as well (3, 4). Matrix metallopeptidase 7 (MMP7), also known as matrilysin, pump-1 protease (PUMP-1), or uterine metalloproteinase, is a member of the MMP family encoded by the *MMP7* gene in humans (5).

As the smallest MMP enzyme, MMP7 has the common characteristics of the MMP family, namely, degradation of casein, fibronectin, gelatin types I, III, IV, and V, and proteoglycans (2). Yet, different from most MMPs, MMP7 possesses its own unique features, which is constitutively enriched in many epithelial cell types, such as the salivary gland epithelium, gallbladder, pancreas, liver, breast, intestine, and reproductive organs (6). More and more evidence has shown that MMP7 plays a critical role in the genesis and development of tumors by reducing cell adhesion, inhibiting cancer cell apoptosis, and inducing vasculogenesis (7, 8). In addition, MMP7 is also considered to be involved in tumor metastasis and inflammatory cascades reaction (9). Recently, MMP7 upregulation has been confirmed in various human cancer types, including lung, melanoma, esophagus, gallbladder, stomach, pancreas, colon, bladder, and other malignancies. Moreover, it was previously demonstrated that the downregulation of MMP7 can inhibit proliferation, migration, and invasion of cancer cells (10).

To our knowledge, however, only one pan-cancer perspective about the whole *MMP* family and its diagnostic/prognostic potential has been published (11). Due to the intricacy of MMP7 in tumorigenesis, it is requisite to conduct a pan-cancer analysis on the correlation between MMP7 and various human cancers based upon big clinical data. Accordingly, the present study applied The Cancer Genome Atlas (TCGA) project and the Gene Expression Omnibus (GEO) database to perform the pan-cancer analysis of MMP7 across all TCGA cancer types considering several aspects, such as gene/protein expression, prognostic value, genetic alteration, immune infiltration, and pathway enrichment analysis.

## Materials and Methods

### TIMER2.0

TIMER2 (tumor immune estimation resource, version 2, http://timer.comp-genomics.org/) is a website for systematical evaluation of immune infiltrates in various cancers (12). In the present study, the “Gene_DE module” was applied to explore the differential expression between tumor tissues and adjacent normal controls for *MMP7* across all TCGA cancer types. In addition, “Immune-Gene module” was used to analyze the relationship between the expression of *MMP7* and immune infiltrates, estimated by multiple immune deconvolution methods, including CIBERSORT, CIBERSORT-ABS, EPIC, MCPCOUNTER, QUANTISEQ, TIMER, and XCELL algorithms, across all TCGA cancers. In our study, macrophages, dendritic cells, and cancer-associated fibroblasts (MΦ, DCs, and CAFs, respectively) were selected. Moreover, the “Gene_Corr” module of TIMER2 was applied to evaluate the heatmap data of the correlation between *MMP7* and some selected genes found in all TCGA tumors. The *P*-values and the correlation (cor) values were acquired using the partial Spearman’s correlation test with the “Purity Adjustment” option.

### GEPIA2

GEPIA2 (Gene Expression Profiling Interactive Analysis, version 2, http://gepia2.cancer-pku.cn/#index) is an updated and enhanced web server for large-scale expression profiling and interactive analysis between tumor and normal tissues from the TCGA and the GTEx (Genotype-Tissue Expression) projects (13). For certain cancer types with only a few or without normal samples, the “Expression Analysis-Box Plot” module of GEPIA2 was used to evaluate and compare the *MMP7* expression between tumor tissues and their paired normal controls of the GTEx database with the setting of “Match TCGA normal and GTEx data”. In addition, a pathological stage analysis across all TCGA cancers was performed using the “Expression Analysis-Pathological Stage Plot” module of GEPIA2 and yielded violin plots of *MMP7* expression. Furthermore, the correlative prognostic analysis of MMP7 across all TCGA tumors was executed *via* the combined application of the “Survival Map” and “Survival Analysis” module of GEPIA2, which was performed using a Kaplan–Meier curve. In addition, the “Expression Analysis-Similar Genes Detection” module of GEPIA2 was used to search for the top 100 *MMP7*-related genes derived from all TCGA tumor tissues and corresponding normal tissues.

### UALCAN

UALCAN (http://ualcan.path.uab.edu/index.html) is a comprehensive web resource for analyzing cancer OMICS data (TCGA, MET500, and CPTAC) (14, 15). In our study, MMP7 protein expression levels in primary tumor and normal tissues obtained from six available datasets of tumors were explored based on the data from the Clinical Proteomic Tumor Analysis Consortium (CPTAC) dataset. Student’s t test was used to calculate the *P*-value with a cutoff of 0.05 (*< 0.05; **< 0.01; ***< 0.001).

### cBioPortal

As an open-access web resource, cBioPortal (www.cbioportal.org) can be used to explore, visualize, and analyze multidimensional cancer genomics data (16, 17). In our study, the “TCGA Pan Cancer Atlas Studies-Cancer Types Summary” module of this website was used to obtain the genetic alterations of MMP7, including the detailed alteration frequencies, mutation types, and copy number alterations (CNA) in all TCGA cancer types. Furthermore, the “Mutations” module was used to acquire the information of the mutated site for MMP7. Next, the “Comparison/Survival” module was used to produce data about the survival differences across all TCGA cancers with or without MMP7 genetic alterations *via* the concurrent generation of Kaplan–Meier curves and log-rank *P*-values.

### String

STRING (https://string-db.org/, version 11.0) is a web server that can predict protein–protein interactions (PPI) (18). This website was searched by inputting MMP7 and *Homo sapiens* with several basic settings: (1) network type-full network, (2) meaning of network edges-evidence, (3) active interaction sources-experiments, (4) minimum required interaction score-low confidence (0.150), and (5) max number of interactors to show no more than 50 interactors in the first shell and custom value with three maximum interactors in the second shell, obtaining 50 experimentally determined MMP7 interacting proteins and the PPI network.

### Metascape

Metascape (http://metascape.org) is a reliable, productive, and intuitive resource for gene annotation and gene list enrichment analysis (19). In this study, a gene list, including the top 100 *MMP7*-correlated genes from the tool of GEPIA2 and the 50 experimentally determined MMP7 interacting genes from String were inputted. Next, *H. sapiens* was inputted as species in step 2, and the “Express Analysis” module was performed in step 3. Finally, the gene list analysis report was exported for the further Kyoto Encyclopedia of Genes and Genomes (KEGG) pathway analysis and gene ontology (GO) enrichment analysis.

### Other Web Servers

OSppc (“https://bioinfo.henu.edu.cn/Protein/OSppc.html”) is a web server named Online consensus Survival analysis web server based on Proteome of Pan-cancers, including TCGA, RPPAs (reverse-phase protein arrays) data, and CPTAC mass spectrometry data. LOGpc (“https://bioinfo.henu.edu.cn/DatabaseList.jsp”), named Long-term Outcome and Gene Expression Profiling Database of pan-cancers, which is a web server encompassing 209 expression datasets, provides different survival terms for distinct cancers. In this study, OSppc was used for pan-cancer differential expression and prognosis analysis at the protein level and LOGpc with multiple data sources including TCGA was utilized for pan-cancer prognosis analysis as well.

TMB and TSI were performed *via* the “Pan-Cancer mutational analysis” module on a Chinese website (“https://www.aclbi.com/static/index.html#/”). The proportional Venn diagram, KEGG pathway, and GO enrichment (based on the data derived from the tool of metascape) dot bubbles and histograms were obtained from another Chinese bioinformatic website (“http://www.bioinformatics.com.cn/”). Both websites are online platforms for data analysis and visualization.

### Immunohistochemistry Staining and Analysis

The present study obtained ethics approval from the Ethics Committee of Affiliated People’s Hospital, Jiangsu University. The validation of MMP7 expression was performed *via* immunohistochemical staining according to the manufacturer’s instructions. Briefly, formalin-fixed paraffin-embedded tissue sections were subject to heat induced epitope retrieval at pH 6.0 for 20 min heating step with the citric buffer. MMP7 antibody was obtained from Proteintech (10374-2-AP, 1:1000 dilution), followed by HRP-labeled secondary antibody (1:5000) and DAB coloring. Images were scanned and analyzed using the Image J software (v1.51n), allowing the quantitation of mean density (integrated optical density (IOD)/area) of the positive staining. GraphPad Prism 6 software was used for the statistical analysis and the results were considered to be significant when *P* value ≤ 0.05, indicated with * in the figure legends (*≤ 0.05; **< 0.01; ***< 0.001; ****< 0.0001).

## Results

### Aberrant Expression of MMP7 in Different Cancers

**Figure 1:**
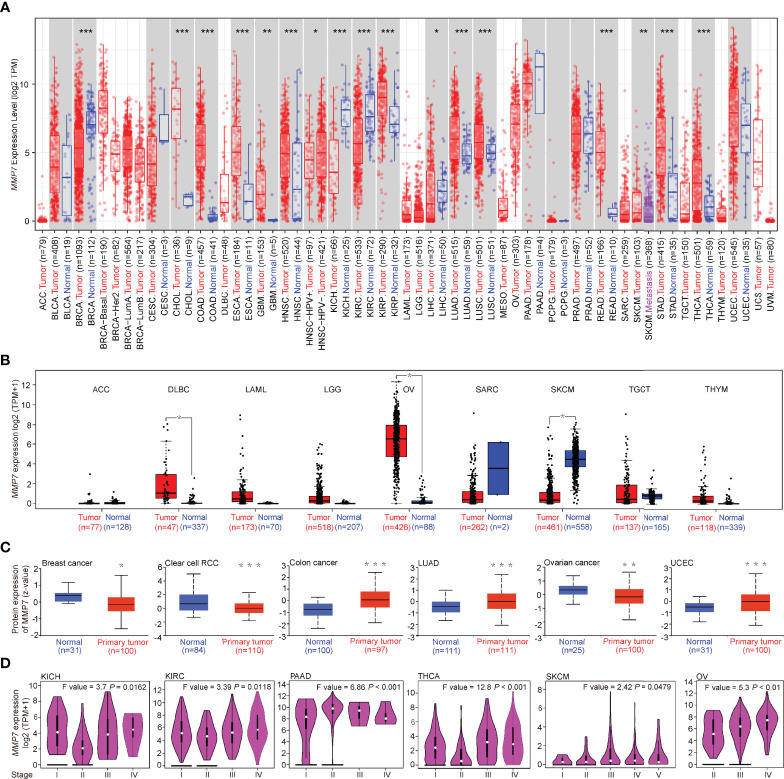
Differential expression of *MMP7* across TCGA cancer types and the pathological stages. **(A)** Matrix metallopeptidase (*MMP7*) expression level in various cancers (red) *vs*. normal controls (blue) using TIMER2. **(B)** The expression difference of *MMP7* between the tumor (red) and normal tissues (blue) in adenoid cystic carcinoma, diffuse large B-cell lymphoma, acute myeloid leukemia, low-grade glioma, ovarian cancer, sarcoma, skin cutaneous melanoma, testicular germ cell tumor, and thymoma (ACC, DLBC, LAML, LGG, OV, SARC, SKCM, TGCT, and THYM, respectively) based on The Cancer Genome Atlas (TCGA) and GTEx database using GEPIA2. **(C)** The protein expression level of MMP7 in tumors tissues (red) compared with the control tissues (blue) of breast cancer, clear cell renal cell carcinoma (RCC), colon cancer, lung adenocarcinoma (LUAD), OV, and uterine corpus endometrial carcinoma (UCEC) was determined based on the CPTAC database by UALCAN. **(D)** Correlation between differential expression of *MMP7* and the pathological stages of kidney chromophobe (KICH), kidney renal clear cell carcinoma (KIRC), pancreatic adenocarcinoma (PAAD), thyroid carcinoma (THCA), KIRC, SKCM, and OV (GEPIA2). *P < 0.05; **P < 0.01; ***P < 0.001.

TIMER2 was used to determine the expression of *MMP7* across various TCGA tumors. As illustrated in **Figure 1A**, the expression of *MMP7* in the tumor tissues of cholangiocarcinoma (CHOL), colon adenocarcinoma (COAD), esophageal carcinoma (ESCA), glioblastoma multiforme (GBM), head and neck squamous cell carcinoma (HNSC), kidney renal papillary cell carcinoma (KIRP), lung adenocarcinoma (LUAD), lung squamous cell carcinoma (LUSC), rectum adenocarcinoma (READ), stomach adenocarcinoma (STAD), and thyroid carcinoma (THCA) was significantly higher than in the normal tissues (*P* < *0,001*). A remarkably higher expression of *MMP7* in the tumor tissue of skin cutaneous melanoma (SKCM) was found compared to metastatic tissue (*P* < 0.01). It is intriguing that the expression level of *MMP7* in the tumor tissues was found to be considerably lower compared to the control tissues in breast invasive carcinoma (BRCA), two pathological types of kidney malignant diseases, namely kidney chromophobe (KICH) and kidney renal clear cell carcinoma (KIRC) with *P* < 0.001, and liver hepatocellular carcinoma (LIHC) with *P* < 0.05.

For certain cancer types without or with limited normal tissues shown in **Figure 1A**, the normal tissues of the GTEx dataset were involved as controls by using GEPIA2. The results indicate that the expression of *MMP7* in the tumor tissues of lymphoid neoplasm diffuse large B-cell lymphoma (DLBC) and ovarian serous cystadenocarcinoma (OV) was considerably higher than the normal controls (**Figure 1B**, *P* < 0.05), while lower *MMP7* expression was detected in SKCM tumor tissue when compared with normal tissue (**Figure 1B**, *P* < 0.05). No significant difference in *MMP7* expression between the tumor tissues and normal tissues in the remaining TCGA cancer types (adrenocortical carcinoma [ACC], bladder urothelial carcinoma [BLCA], and others) was determined based on whatever TCGA dataset or TCGA plus GTEx dataset was selected (**Figures 1A, B**).

The results of the CPTAC dataset revealed that the protein expression level of MMP7 is notably higher in the primary tumor tissues of colon cancer, LUAD, and uterine corpus endometrial carcinoma (UCEC) than in the normal tissues (**Figure 1C**, *P* < 0.001). The protein expression level of MMP7 was also found to be significantly lower in the primary tumor tissues of breast cancer (**Figure 1C**, *P* < 0.05) and clear cell RCC (**Figure 1C**, *P* < 0.001) when compared with normal tissues. Yet, MMP7 expression level was significantly lower in ovarian cancer tumor tissues (**Figure 1C**, *P* < 0.01) when compared with normal tissue, which is opposite to the result shown in **Figure 1B**.

To further explore pan-cancer differential expression of MMP7 at the protein level, OSppc for pan-cancer differential expression was applied. The results were almost consistent with what is illustrated in **Figure 1C**. Besides, the OSppc database also showed that there was no significant difference in protein expression of MMP7 between the tumor tissues and normal tissues in GBM (*P* = 0.1003, figure not shown).

**Figure 2:**
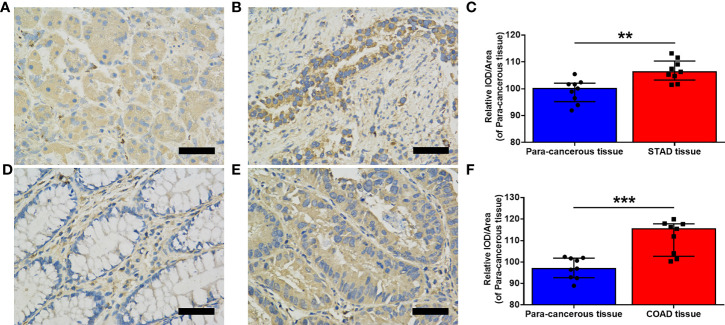
Evaluation of MMP7 expression in stomach adenocarcinoma (STAD) and colon adenocarcinoma (COAD). **(A, B)** Representative immunohistochemistry (IHC) stained for MMP7 in the para-cancerous tissue **(A)** and tumor tissue **(B)** of the stomach, respectively. **(C)** Quantitation of MMP7 expression in the para-cancerous tissue and STAD tissue shown as mean density (integrated optical density (IOD)/area) plotted as means ± SD (n = 9). **(D, E)** Representative IHC stained for MMP7 in the para-cancerous tissue **(D)** and tumor tissue **(E)** of the colon, respectively. **(F)** Quantitation of MMP7 expression in the para-cancerous tissue and COAD tissue shown as mean density (IOD/area) plotted as means ± SD (n=9). Scale bar=50 μm. **P < 0.01; ***P < 0.001.

Moreover, immunohistochemistry staining was performed using nine pairs of matched cancerous and para-cancerous tissues in STAD and COAD, respectively, to verify the expression of MMP7. As illustrated in **Figure 2**, the expression of MMP7 was significantly higher in STAD tumor tissue (**Figure 2B**) when compared with the para-cancerous tissue (**Figure 2A**) (*P* < 0.01, **Figure 2C**), findings that are consistent with online data from TCGA and CPTAC datasets. Similar results were obtained from the matched cancerous tissue (**Figure 2E**) and para-cancerous tissue (**Figure 2D**) in COAD (*P* < 0.001, **Figure 2F**). Furthermore, the clinicopathological information of the above-mentioned STAD and COAD patients is summarized in **Supplementary Table 1**.

By using the “Expression Analysis-Pathological Stage Plot” module of GEPIA2, a significant association between *MMP7* expression and the different pathological stages in several cancer types (**Figure 1D**), including KICH (Stage I *vs*. Stage II, *P* < 0.05), KIRC (Stage I *vs.* Stage II, *P* < 0.05), pancreatic adenocarcinoma (PAAD) (Stage I *vs*. Stage II, *P* < 0.001), THCA (Stage I *vs*. Stage II, *P* < 0.001), SKCM (Stage I *vs*. Stage II, *P* < 0.05), and OV (Stage II *vs*. Stage III, *P* < 0.01), was detected.

### Prognostic Analysis of MMP7 Across All TCGA Cancers

**Figure 3:**
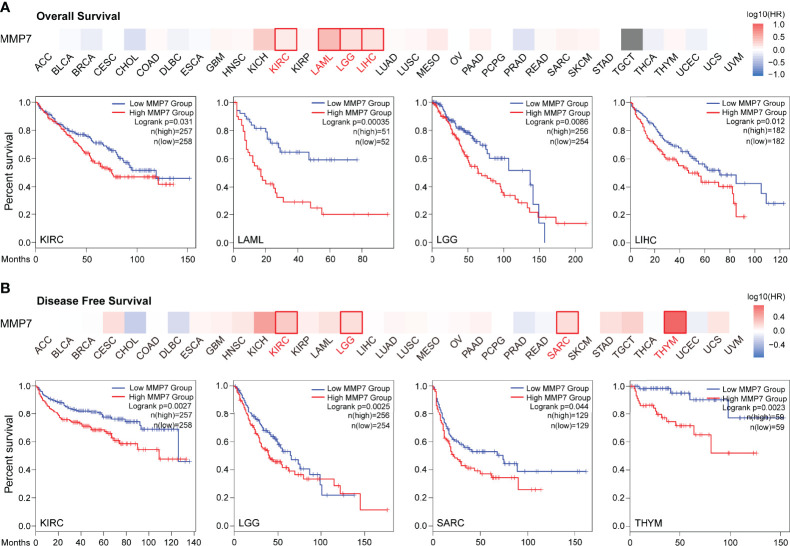
Survival analysis of *MMP7* in different TCGA cancers (GEPIA2). The heatmap and Kaplan–Meier curve of overall survival (OS) **(A)** and disease-free survival (DFS) **(B)** shows that high expression of *MMP7* indicates poor prognosis in KIRC, LAML, LGG, liver hepatocellular carcinoma (LIHC), KIRC, LGG, SARC, and THYM.

To evaluate the prognostic value of differential expression of *MMP7* in all TCGA tumors, the correlation between *MMP7* expression and survival data was determined *via* GEPIA2. As presented in **Figure 3A**, high expression of *MMP7* was significantly associated with poorer overall survival (OS) in KIRC (*P* = 0.031), acute myeloid leukemia (LAML) (*P* = 0.00035), brain lower grade glioma (LGG) with *P* = 0.0086, and LIHC (*P* = 0.012). As for the disease-free survival (DFS) analysis, low expression of *MMP7* was found indicating remarkably better prognosis in KIRC (*P* = 0.0027), LGG (*P* = 0.0025), sarcoma (SARC) (*P* = 0.044), and thymoma (THYM) with *P* = 0.0023 (**Figure 3B**). Additionally, a meta-analysis (Cox regression analysis) was performed to assess the prognostic value of the *MMP7* in different cancer types based on the online platform “https://xenabrowser.net/datapages/”. As shown in **Supplementary Figure S1**, high expression of *MMP7* correlated remarkably with poor OS in LIHC (*P* < 0.001), PAAD (*P* = 0.03), KIRC (*P* = 0.03), LAML (*P* < 0.001), and LGG (*P* = 0.022), findings that are almost consistent with the results in **Figure 2A**.

Moreover, OSppc for pan-cancer prognosis analysis was used to evaluate the prognostic role of MMP7 in different cancer types at the protein level. As shown in **Supplementary Figure S2**, lower protein expression of MMP7 was significantly associated with better OS in KIRC (*P* = 0.0444), which was parallel with the prognostic value of *MMP* gene expression in KIRC (**Figure 3A**).

At the same time, LOGpc with multiple data sources including TCGA was utilized for pan-cancer prognosis analysis of *MMP7*. It was demonstrated that higher expression of *MMP7* was associated with better OS in DLBC (**Supplementary Figure S3A**) (GSE57611, *P* = 0.0353) and OV (**Supplementary Figure S3J**) (GSE17260, *P* = 0.0038). **Supplementary Figure S3** also revealed that high expression of *MMP7* significantly indicated poor OS in gastric cancer (B) (combined data sources, *P* = 2e-04), gastric cancer (C) (GSE62254, *P* = 0.0301), gastric cancer (D) (GSE84437, *P* = 0.0302), gastric cancer (E) (TCGA, *P* = 0.0099), KIRP (F) (TCGA, *P* = 0.0173), LIHC (G) (TCGA, *P* = 0.0171), lung cancer (LUCA) (H) (GSE5123, *P* = 0.0115), LUCA (I) (GSE31210, *P* = 0.0341), OV (K) (GSE23554, *P* = 0.0261), and PAAD (K) (GSE62452, *P* = 0.0334). Additionally, the relationship between differential expression of MMP7 and the potential clinical significance across all the TCGA cancer types is summarized in **Supplementary Table S2**.

### Genetic Alteration Analysis of MMP7

**Figure 4:**
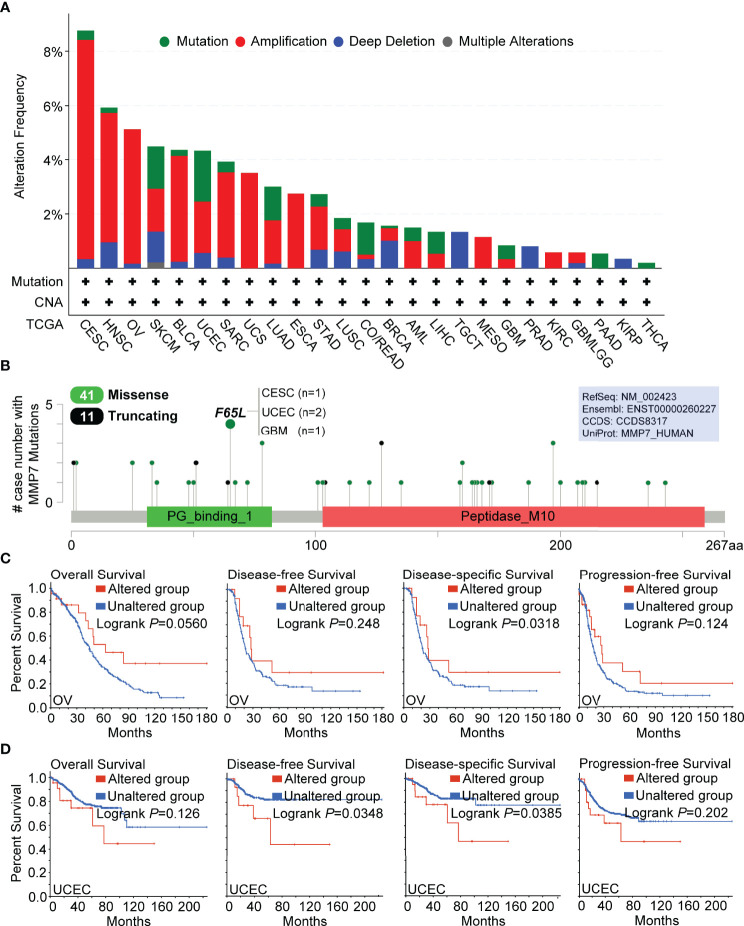
Genetic alteration analysis of MMP7 by cBioPortal. **(A)** Summary of the alteration frequency for different mutation types in various cancers. **(B)** Presentation of the types, sites, and case number of MMP7 genetic alteration. The prognostic value of MMP7 genetic alteration was analyzed and the Kaplan–Meier curves of OS, DFS, disease-specific survival, and progression-free survival in OV **(C)** and UCEC **(D)** are demonstrated.

Genetic alteration analysis of MMP7 was performed using cBioPortal. The results demonstrated that cervical squamous cell carcinoma and endocervical adenocarcinoma (CESC) had the highest alteration frequencies involving MMP7, which was found to be around 8% with “amplification” as the elementary alteration type (**Figure 4A**). Additionally, the “amplification” type of copy number alterations (CNA) was the main type found in several cancers, including HNSC (~5% frequency), OV (~5% frequency), BLCA (~4% frequency), and SARC (~3% frequency). Moreover, all uterine carcinosarcoma (UCS), ESCA, mesothelioma (MESO), and KIRC cases with genetic alterations had amplification of MMP7, with frequencies around 4%, 3%, 1%, and < 1%, respectively (**Figure 4A**). All testicular germ cell tumors (TGCT) with a ~1.5% frequency, prostate adenocarcinoma (PRAD) with a ~1% frequency, and KIRP (< 1% frequency) cases with genetic alterations were found to hold the copy number deletion of MMP7 as their alteration type (**Figure 4A**).

**Figure 4B** illustrates the detailed types, sites, and case numbers for the genetic MMP7 alterations and also showed that the cardinal type of genetic MMP7 alteration is the missense mutation. Furthermore, the F65L mutation located at the peptidoglycan binding domain (PG_binding_1 domain containing 31–82 aa) was associated with the maximum cases with MMP7 mutations, which was detected in one case of CESC, two cases of UCEC, and one case of GBM (**Figure 4B**).

Furthermore, the potential correlation between the genetic alteration of MMP7 and the prognosis across all TCGA cancer types was determined. It was found that OV cases with altered MMP7 indicated better clinical outcomes in disease-specific survival (*P* = 0.0318) but not OS (*P* = 0.056), DFS (*P* = 0.248), and progression-free survival (*P* = 0.124) when compared with the unaltered group (**Figure 4C**). **Figure 4D** reveals that UCEC cases with unaltered MMP7 demonstrated poorer outcome with respect to DFS (*P* = 0.0348) and disease-specific survival (*P* = 0.0385) but not OS (*P* = 0.126) and progression-free survival (*P* = 0.202) than the unaltered group. For the other TCGA cancers (except KIRC with limited cases and only one case of censoring data in the altered MMP7 group), no significant difference in the prognostic data between the altered and unaltered MMP7 groups was detected (data not shown).

**Figure 5:**
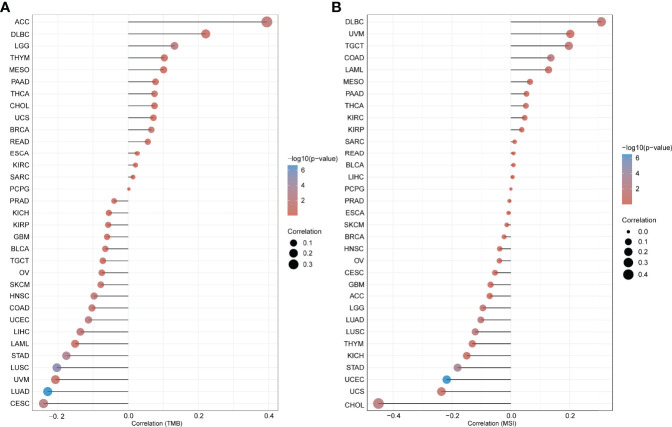
Correlation between the expression of *MMP7* and tumor mutational burden (TMB)/microsatellite instability (MSI). Spearman’s correlation analysis of the tumor mutational burden (TMB) **(A)** microsatellite instability (MSI) **(B)** and *MMP7* gene expression. The horizontal axis represents the correlation coefficient between *MMP7* and TMB/MSI, the ordinate is the different cancer types, the size of the dots represents the size of the correlation coefficient, and the different colors represent the significance of the *P*-value.

Additionally, the association between the expression of *MMP7* and tumor mutational burden/microsatellite instability (TMB/MSI) was explored *via* the open-access platform, “http://www.bioinformatics.com.cn/”, based on the R software v4.0.3. As illustrated in **Figure 5A**, a positive association between *MMP7* expression and TMB was indicated for LGG (*P* = 0.0052), ACC (*P* = 0.021), and BRCA (*P* = 0.036), yet a negative correlation was indicated for LUAD (*P* = 2.19^−07^), LUSC (*P* =1.02^−05^), STAD (*P* = 0.00069), CESC (*P* = 0.0032), UCEC (*P* = 0.01), LIHC (*P* = 0.02), and HNSC (*P* = 0.0032). **Figure 5B** indicates that the expression of *MMP7* is significantly and positively correlated with MSI in COAD (*P* = 0.005), TGCT (*P* = 0.028), and DLBC (*P* = 0.032) but negatively associated with MSI in UCEC (*P* = 3.25^−07^), STAD (*P* = 0.00043), CHOL (*P* = 0.0064), LUSC (*P* = 0.0073), LUAD (*P* = 0.021), and LGG (*P* = 0.043).

### Immune Cell Infiltration of MMP7 in TCGA Cancer Types

**Figure 6:**
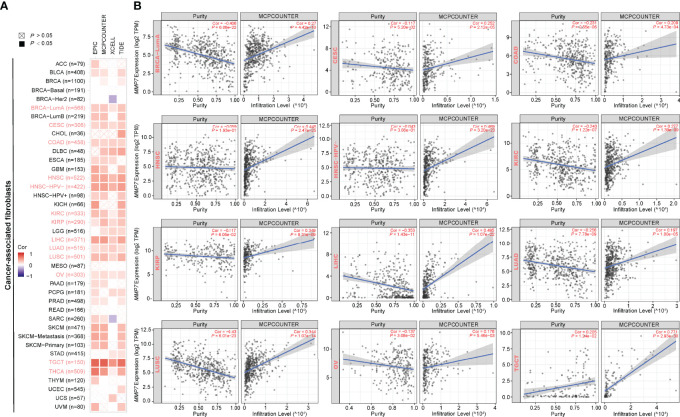
Correlation between the expression of *MMP7* and cancer-associated fibroblasts (CAFs) infiltration (TIMER2). **(A)** The heatmap of purity-adjusted Spearman’s correlation analysis between *MMP7* gene expression and the infiltration levels of CAFs across TCGA cancers based on EPIC, MCPCOUNTER, XCELL, and TIDE algorithms. The red color indicates positive correlation while the purple color indicates negative correlation. **(B)** The scatter plots representing the relationship between CAFs infiltrates estimation value and *MMP7* gene expression by the method of MCPCOUNTER are shown, in BRCA-LumA, CESC, COAD, HNSC, HNSC-HPV-, KIRC, KIRP, LIHC, LUAD, LUSC, OV, TGCT, and THCA.

The primary role of MMP7 is to facilitate degradation of the extracellular matrix (ECM) (2), which constitutes the major components of the tumor microenvironment (TME) together with blood vessels, signaling molecules, immune cells, and fibroblasts (20, 21). TME and cancer cells and cross-talk between them play a critical role in cancer development and progression (22). Hence, the potential correlation between the expression of *MMP7* and the infiltration levels of CAFs/tumor-infiltrating immune cells across all TCGA cancers using EPIC, MCPCOUNTER, XECLL, TIDE, CIBERSORT, CIBERSORT-ABS, and QUANTISEQ methods was explored. As shown in **Figure 6A**, a considerably positive correlation between the expression of *MMP7* and the infiltration of CAFs was indicated in BRCA-LumA, CESC, COAD, HNSC, human papillomavirus (HNSC-HPV), KIRC, KIRP, LIHC, LUAD, LUSC, OV, TGCT, and THCA based on all EPIC, MCPCOUNTER, XECLL, and TIDE algorithms. **Figure 6B** illustrates the specific scattergrams for the above-mentioned tumor types derived from the MCPCOUNTER algorithm, which was applied as an example in the present study (data not shown for EPIC, XECLL, and TIDE algorithms).

**Figure 7:**
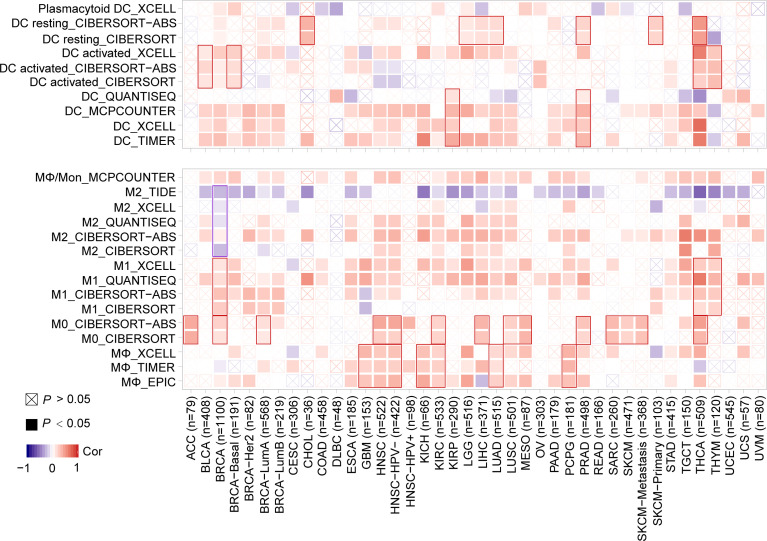
Correlation between *MMP7* expression and the infiltration level of dendritic cell/macrophage (TIMER2). The figure presents the heatmaps of purity-adjusted Spearman’s correlation analysis between *MMP7* gene expression and the infiltration levels of dendritic cell/macrophage in diverse cancer types using EPIC, MCPCOUNTER, XECLL, TIDE, CIBERSORT, CIBERSORT-ABS, and QUANTISEQ methods.

Furthermore, a significantly positive correlation between the expression of *MMP7* and the infiltration of dendritic cells (DCs) in KIRP and PRAD, between *MMP7* expression and infiltration of activated-DC in BLCA, BRCA-basal, THCA, and THYM, between *MMP7* expression and resting-DC infiltration for CHOL, LGG, LIHC, LUAD, PRAD, SKCM-Primary, and THCA was found (**Figure 7**). **Figure 7** also shows a statistically positive association between the expression of *MMP7* and infiltration of MΦ in GBM, HNSC, HNSC-HPV-, KICH, KIRC, LUAD, and pheochromocytoma and paraganglioma (PCPG), between *MMP7* expression and the infiltration of MΦ in ACC, BRCA, BRCA-LumA, HNSC, HNSC-HPV-, KIRC, LIHC, LUSC, MESO, PRAD, SARC, SKCM, and SKCM-metastasis, and between *MMP7* expression and M1 for BRCA, TGCT, and THYM. Interestingly, a significantly negative correlation of the expression of *MMP7* and the infiltration level of M2 only in BRCA (**Figure 7**) was noted. As for the other types of tumor-infiltrating immune cells (such as CD8+ T and CD4+ T cells), no consistent results between *MMP7* expression and the infiltration value of them were obtained (data not shown).

### Functional Enrichment Analysis of MMP7-Related Genes

**Figure 8:**
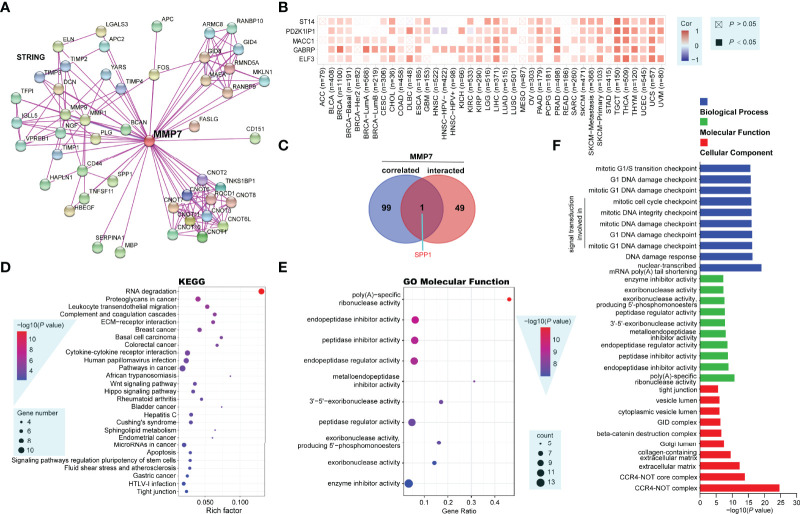
Functional enrichment analysis of MMP7-related genes (String, Metascape and other open-access web servers). **(A)** Protein–protein interaction network of 50 experimentally determined MMP7 interacting genes by the web server of String. **(B)** The heatmap showing the correlation analysis between the expression of *MMP7* and ELF3, GABRP, MACC1, PDZK1IP1, and ST14 in different cancer types. **(C)** The Venn diagram presenting the intersection analysis of MMP7-interacting and *MMP7*-correlated genes. **(D)** The dot bubble of the Kyoto Encyclopedia of Genes and Genomes (KEGG)-enriched terms based on MMP7-interacting and *MMP7*-related genes, in which the most significant was marked in red. **(E)** The dot bubble of gene ontology (GO) enrichment analysis in molecular function terms with the most significance marked in red. **(F)** Bar plot of typical enriched GO terms including biological process (blue), molecular function (green), and cellular component (red), the top 10 GO terms in each category are illustrated according to the *P*-values.

To explore the potential molecular mechanisms of *MMP7* in tumorigenesis, MMP7-interacting genes and *MMP7*-correlated genes were screened out using the STRING and GEPIA2 tools, respectively, for the pathway and process enrichment analysis. **Figure 8A** illustrates a total of 50 experimentally determined MMP7 interacting genes in the PPI network. Next, the top 100 *MMP7*-correlated genes, among which five genes (E74 Like ETS Transcription Factor 3 [ELF3] with R = 0.61, Gamma-Aminobutyric Acid Type A Receptor Subunit Pi [GABRP] with R = 0.57, MET Transcriptional Regulator MACC1 [MACC1] with R = 0.57, PDZK1 Interacting Protein 1 [PDZK1IP1] with R = 0.6, and ST14 Transmembrane Serine Protease Matriptase [ST14] with R = 0.56) ranked by the top 5 R values were listed. The maximum positive correlation coefficients were chosen to determine the corresponding heatmap, indicating a positive correlation between *MMP7* expression and the five genes described above in a large proportion of all the TCGA cancers (**Figure 8B**). Furthermore, the above two groups of genes were performed for the intersection analysis, and one common gene, Secreted Phosphoprotein 1 (SPP1), was obtained and presented in a proportional Venn diagram (**Figure 8C**).

The above two groups of gene datasets were further utilized to perform KEGG and GO enrichment analysis using Metascape and another open platform, “http://www.bio-informatics.com.cn/”. The dot bubble of the KEGG enrichment analysis showed all of the potential pathways implicated in the effects of MMP7 on tumorigenesis in which “RNA degradation” might be a particular pathway with the most significant *P*-value and the maximal gene counts (**Figure 8D**). The GO (molecular function) enrichment analysis data of **Figure 8E** listed the top 10 cellular biological functions ranked by their *P*-values, including poly(A)-specific ribonuclease, endopeptidase inhibitor, peptidase inhibitor, endopeptidase regulator, metalloendopeptidase inhibitor, 3’-5’-exoribonuclease, peptidase regulator, exoribonuclease (producing 5’-phosphomonoesters), exoribonuclease, and enzyme inhibitor activities. In addition, typical enriched GO terms consisting of three categories, namely, biological process (blue), molecular function (green), and cellular component (red) are demonstrated in **Figure 8F**. Each category showed the top 10 GO terms according to the *P*-values.

## Discussion and Conclusions

MMP7 is a well-established and important participant in regulating various pathophysiological processes, including wound healing, aging, bone growth, remodeling, and signal transduction pathways that control inflammation, cell growth, and angiogenesis (7, 23–25). Besides, MMP7 has been recognized as an oncogenic factor that manages the oncogenesis and progression of multiple tumors by mediating differentiation, proliferation, invasion, and metastasis of cancer cells (10). In this study, a comprehensive pan-cancer analysis of MMP7 across all the TCGA cancers to uncover the potential roles and underlying mechanism of MMP7 in the occurrence, development, and clinical outcomes of different cancers is presented.

Consistent with previous studies, heterogeneous expression of *MMP7* across all TCGA cancers with downregulation in BRCA, KIRC, KICH, and LIHC and upregulation in most of the other cancers was obtained (10, 11), while low expression of *MMP7* was obtained in SKCM tumor tissue, a finding that was opposite to that in a previous study (26). Larger sample sizes and the setting of “Match TCGA normal and GTEx data” in this study might contribute to the different results. Due to the limitation of the CPTAC dataset, only MMP7 protein expression in breast cancer, clear cell RCC, colon cancer, LUAD, ovarian cancer, and UCEC was obtained. Parallel to the *MMP7* gene expression level, the MMP7 protein was downregulated in breast cancer and clear cell RCC. Abundant expression of MMP7 protein was detected in colon cancer, LUAD, and UCEC in this study, a finding that was similar to those from previous studies (27–30). Interestingly, the results concerning the overexpression of *MMP7* gene in ovarian cancer shown in **Figure 1B** was not reproduced at the protein level as shown in **Figure 1C**. Previous studies have also demonstrated that gene expression analysis of *MMP7* was inconsistent with its protein expression (31). The opposite results illustrated in **Figures 1B, C** were most likely based on the different databases and inconsistent sample sizes. MMP7 exhibits elevated expression in multiple cancer types, including OV (31, 32). On that account, the expression of MMP7 in OV at both the gene and protein levels needs to be confirmed in a larger series in further studies. The correlation between the expression of *MMP7* and the pathological stages in different cancer types was evaluated first. As the tumor progressed, *MMP7* expression increased, suggesting that MMP7 plays an essential role in the tumorigenesis and development of KICH, KIRC, PAAD, THCA, SKCM, and OV.

KIRC, LAML, LGG, and LIHC patients with high expression of *MMP7* were significantly correlated with worse OS; KIRC, LGG, SARC, and THYM patients with high expression of *MMP7* were remarkably associated with worse DFS. LOGpc analysis with multiple data sources also proved that high expression of *MMP7* indicated poor OS in STAD, KIRP, LIHC, LUCA, and PAAD. Overexpression of *MMP7* is always correlated with poor prognosis, which has been demonstrated in many previous studies for gastric cancer (33, 34), pancreatic cancer (35, 36), colorectal cancer (37), bladder cancer (38), ovarian cancer (32), and others. In the present study, different results addressing the prognostic value of *MMP7* expression in various cancer types were obtained, which might have stemmed from larger sample sizes and multiple datasets. Overall, these results suggest that MMP7 could function as an adverse prognostic factor for different cancers.

Defined as a copy number increase of a restricted region of a chromosome arm, gene amplification is a typical alteration in cancer (39, 40). Moreover, gene amplification is considered to be a major cause of tumorigenesis (40). In our study, gene amplification was detected in most TCGA cancer types and presented the highest alteration frequency. According to the Pfam database, the protein sequence of MMP7_Human (consists of 267 amino acids) has two major domains, PG_binding_1 and Peptise_M10. It was shown that the primary type of genetic alteration of MMP7 in tumors is the missense mutation in which F65L mutation located at PG_binding_1 domain has the maximum number of cases containing the MMP7 mutation. Furthermore, the F65L mutation appears to be capable of inducing a frame shift mutation of the *MMP7* gene, causing the translation from the amino acid F (Phenylalanine) to L (Leucine) at the 65th site of MMP7 protein sequence, which could be a risk factor of oncogenesis in CESC, UCEC, and GBM. However, this finding still needs further investigation. We then focused on the prognostic value of genetic MMP7 alterations in different cancers. Altered MMP7 is correlated with better disease-specific survival in OV yet is associated with worse DFS and disease-specific survival in UCEC, findings that suggest that genetic alterations of MMP7 may play a significant role in different cancers. Nevertheless, extremely limited studies showing the relationship between the genetic alteration of MMP7 and clinical outcomes in various cancers have been published.

It is clear that TMB is a promising biomarker for prediction of sensitivity to immune checkpoint inhibitor treatments in several cancer types, especially in cancers with MSI (41, 42). The potential correlation between the expression of *MMP7* and TMB/MSI across various cancers in the present study was assessed. According to the results, MMP7 might be a potential target for cancer immune therapy in LGG, ACC, BRCA, LUAD, LUSC, STAD, CESC, UCEC, LIHC, HNSC, COAD, TGCT, DLBC, and CHOL. However, the influence of the expression of *MMP7* on TMB/MSI has rarely been reported previously. Accumulating evidence indicates that CAFs, tumor-associated immune cells, and ECMs in TME can interact with cancer cells thus playing essential roles in tumor growth, invasion, antitumor immunotherapy, and prognosis (10, 43–45). Hence, the correlation between *MMP7* expression and cancer immunity was assessed. It was shown that the expression of *MMP7* positively correlated with the infiltration of CAFs in most TCGA cancer types and was positively associated with the infiltration of DC/activated–DC/resting DCs and MΦ/M0/M1 in some specific tumor types, indicating that MMP7 may also reflect the immune status in different cancers. Of note, *MMP7* expression positively correlated with M0 and M1 yet negatively correlated with M2 in BRCA, implying that *MMP7* overexpression may cause a shift toward an M1-like phenotype. Previous studies have shown that tumor-associated macrophages in TME predominately resemble the M2-polarized cells and correlate with poor prognosis (46, 47). Thus, *MMP7* expression may contribute to better clinical outcomes. In-depth investigations are still needed to further investigate the association between the *MMP7* expression and tumor-associated macrophage infiltration and the underlying molecular mechanisms.

Nowadays, with the development of computational biology and bioinformatics, multiple computational methods, especially KEGG pathways and GO terms, have been widely applied to describe specific pathways and biological processes in different cancers (48, 49). In the present study, 50 MMP7-interacting genes and 100 *MMP7*-correlated genes were first obtained using STRING and GEPIA2, respectively. Functional enrichment analysis using KEGG pathways and GO terms methods with the 150 genes was then performed. It was demonstrated that these genes may play important roles in the “RNA degradation”, “poly(A)-specific ribonuclease activity”, “cell-cycle checkpoint”, and other pathways. Previous studies have shown that RNA degradation is a process during the cell cycle that regulates RNA-dependent mechanisms, with ribonucleases (endonucleases and exonucleases) along with other enzymes and cofactors that are also involved (50, 51). Moreover, both altered RNA processing and DNA damage checkpoints are considered to be strongly linked to pathogenesis of human cancers (52–54). Taken together, these data suggest that RNA processing and DNA damage checkpoints might implicate the functional mechanisms of MMP7 and that MMP7 might be a potential therapeutic drug target in different cancers.

To summarize, our study systemically analyzed MMP7 in a pan-cancer manner, evaluating the potential association of the expression of MMP7 with the clinical outcome (**Supplementary Table S2**), pathological stages, TMB, MSI, and CAFs/immune cell infiltration in various cancer types, which will assist in providing a clearer panorama for the roles of MMP7 across human cancers.

## Data Availability Statement

The datasets presented in this study can be found in online repositories. The names of the repository/repositories and accession number(s) can be found in the article/**Supplementary Material**.

## Ethics Statement

The studies involving human participants were reviewed and approved by Affiliated People’s Hospital, Jiangsu University. The patients/participants provided their written informed consent to participate in this study.

## Author Contributions

NM and YL undertook the data analysis and wrote the manuscript. LX, PJ, and XB designed the study and edited the manuscript. JD and YW aided in the methodology and data duration. XZ, FY, YZ, and JZ assisted in the conception and data representation. NM and LX contributed to the funding. All of the authors reviewed the data and analysis, and read and approved the manuscript.

## Funding

Database mining, data collection, and manuscript preparation of this work was supported by the funding from the Innovation and Entrepreneurship Plan of Jiangsu Province (No. SCBS202101) and the Affiliated People’s Hospital of Jiangsu University (No. KFB2020005) from LX. Figure editing and layout and language editing service of this work were supported by the Medical and Health Guidance Project of Xiamen City (3502Z20214ZD1219) from NM.

## Conflict of Interest

The authors declare that the research was conducted in the absence of any commercial or financial relationships that could be construed as a potential conflict of interest.

## Publisher’s Note

All claims expressed in this article are solely those of the authors and do not necessarily represent those of their affiliated organizations, or those of the publisher, the editors and the reviewers. Any product that may be evaluated in this article, or claim that may be made by its manufacturer, is not guaranteed or endorsed by the publisher.

## Supplementary Material

Supplementary Figure 1 Assessment of the prognostic value of MMP7 in different cancer types by a meta-analysis. High expression of MMP7 indicated poor overall survival in LIHC (*P* < 0.001), PAAD (*P* = 0.03), KIRC (*P* = 0.03), LAML (*P* < 0.001) and LGG (P=0.022) were demonstrated.

Supplementary Figure 2 Evaluation of the prognostic role of MMP7 in kidney renal clear cell carcinoma (KIRC) at the protein level based on the online platform “OSppc” (https://bioinfo.henu.edu.cn/Protein/OSppc.html). Low protein expression of MMP7 correlated with better overall survival in KIRC (*P* = 0.0444).

Supplementary Figure 3 Survival analysis of *MMP7* in different malignancies based on the web sever of LOGpc with multiple data sources. Higher expression of *MMP7* indicated better overall survival in DLBC (A, GSE57611) and OV (J, GSE17260). Poor overall survival correlated with high expression of MMP7 in gastric cancer (B, combined data sources), gastric cancer (C, GSE62254), gastric cancer (D, GSE84437), gastric cancer (E, TCGA), KIRP (F, TCGA), LIHC (G, TCGA), LUCA (H, GSE5123), LUCA (I, GSE31210), OV (K, GSE23554) and PAAD (K, GSE62452).

## Glossary

**Table:**

ACC	adrenocortical carcinoma
BLCA	bladder urothelial carcinoma
BRCA	breast invasive carcinoma
CAFs	cancer-associated fibroblasts
CESC	cervical squamous cell carcinoma and endocervical adenocarcinoma
CAN	copy number alteration
CHOL	cholangiocarcinoma
COAD	colon adenocarcinoma
CPTAC	Clinical Proteomic Tumor Analysis Consortium
DC	dendritic cell
DFS	disease-specific survival
DLBC	diffuse large B-cell lymphoma
ECM	extracellular matrix
ELF3	E74 Like ETS Transcription Factor 3
ESCA	esophageal carcinoma
GABRP	Gamma-Aminobutyric Acid Type A Receptor Subunit Pi
GBM	glioblastoma multiforme
GEO	Gene Expression Omnibus
GEPIA2	Gene Expression Profiling Interactive Analysis version 2
GO	gene ontology
GTE	genotype-tissue expression
HNSC	head and neck squamous cell carcinoma
KEGG	Kyoto Encyclopedia of Genes and Genomes
KICH	kidney chromophobe
KIRC	kidney renal clear cell carcinoma
KIRP	kidney renal papillary cell carcinoma
LAML	Acute myeloid leukemia
LGG	lower grade glioma
LIHC	liver hepatocellular carcinoma
LUAD	lung adenocarcinoma
LUSC	lung squamous cell carcinoma
MΦ	macrophage
MACC1	MET Transcriptional Regulator MACC1
MESO	mesothelioma
MMP	matrix metalloproteinase
MSI	microsatellite instability
OV	ovarian serous cystadenocarcinoma
OS	overall survival
PAAD	pancreatic adenocarcinoma
PCPG	pheochromocytoma and paraganglioma
PDZK1IP1	PDZK1 Interacting Protein 1
PPI	protein-protein interactions
PRAD	prostate adenocarcinoma
READ	rectum adenocarcinoma
SARC	sarcoma
SKCM	skin cutaneous melanoma
SPP1	secreted phosphoprotein 1
ST14	ST14 transmembrane serine protease matriptase
STAD	stomach adenocarcinoma
TCGA	The Cancer Genome Atlas
TGCT	testicular germ cell tumor
THCA	thyroid carcinoma
THYM	thymoma
TIMER2	tumor immune estimation resource version 2
TMB	tumor mutational burden
TME	tumor microenvironment
UCEC	uterine corpus endometrial carcinoma
UCS	uterine carcinosarcoma
UVM	uveal melanoma.
