# Supplementary material for: A Comprehensive Pan-Cancer Analysis of the Tumorigenic Role of Matrix Metallopeptidase 7 (MMP7) Across Human Cancers
Source: Front Oncol. 2022 Jun 17;12:916907. doi: 10.3389/fonc.2022.916907 (PMC9248742; doi:10.3389/fonc.2022.916907)
Supplement: Supplementary file 1 [file DataSheet_1.docx]

**Supplementary Materials and Methods**

**Meta-analysis**

The online platform “https://xenabrowser.net/datapages/” were systematically searched to identify related datasets on the association between MMP7 and prognosis of different cancer types. The meta-analysis (Cox regression analysis) was utilized to determine the prognostic significance of MMP7 in tumor patients. Combined hazard ration (HR) and 95% CI were calculated to evaluate the correlation of MMP7 expression with its prognosis, in which higher HR indicates worse prognosis. P values ≤ 0.05 were considered significant. Statistical analysis was performed by using R software v4.0.3.
