# Supplementary figures and images for: A Comprehensive Pan-Cancer Analysis of the Tumorigenic Role of Matrix Metallopeptidase 7 (MMP7) Across Human Cancers
Source: Front Oncol. 2022 Jun 17;12:916907. doi: 10.3389/fonc.2022.916907 (PMC9248742; doi:10.3389/fonc.2022.916907)

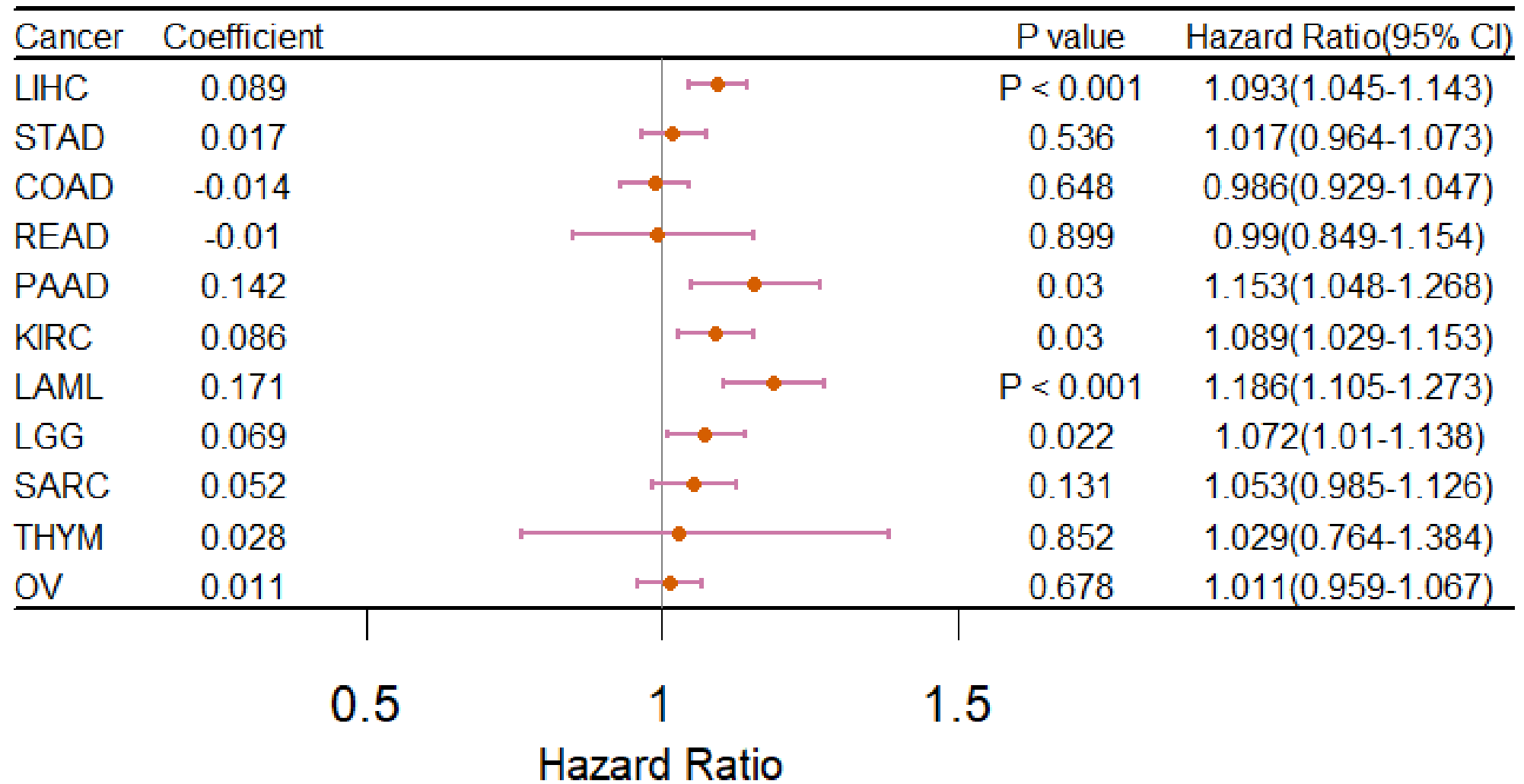

Supplement: Supplementary Figure 1 — Assessment of the prognostic value of MMP7 in different cancer types by a meta-analysis. High expression of MMP7 indicated poor overall survival in LIHC (P < 0.001), PAAD (P = 0.03), KIRC (P = 0.03), LAML (P < 0.001) and LGG (P=0.022) were demonstrated. [file DataSheet_2.pdf]

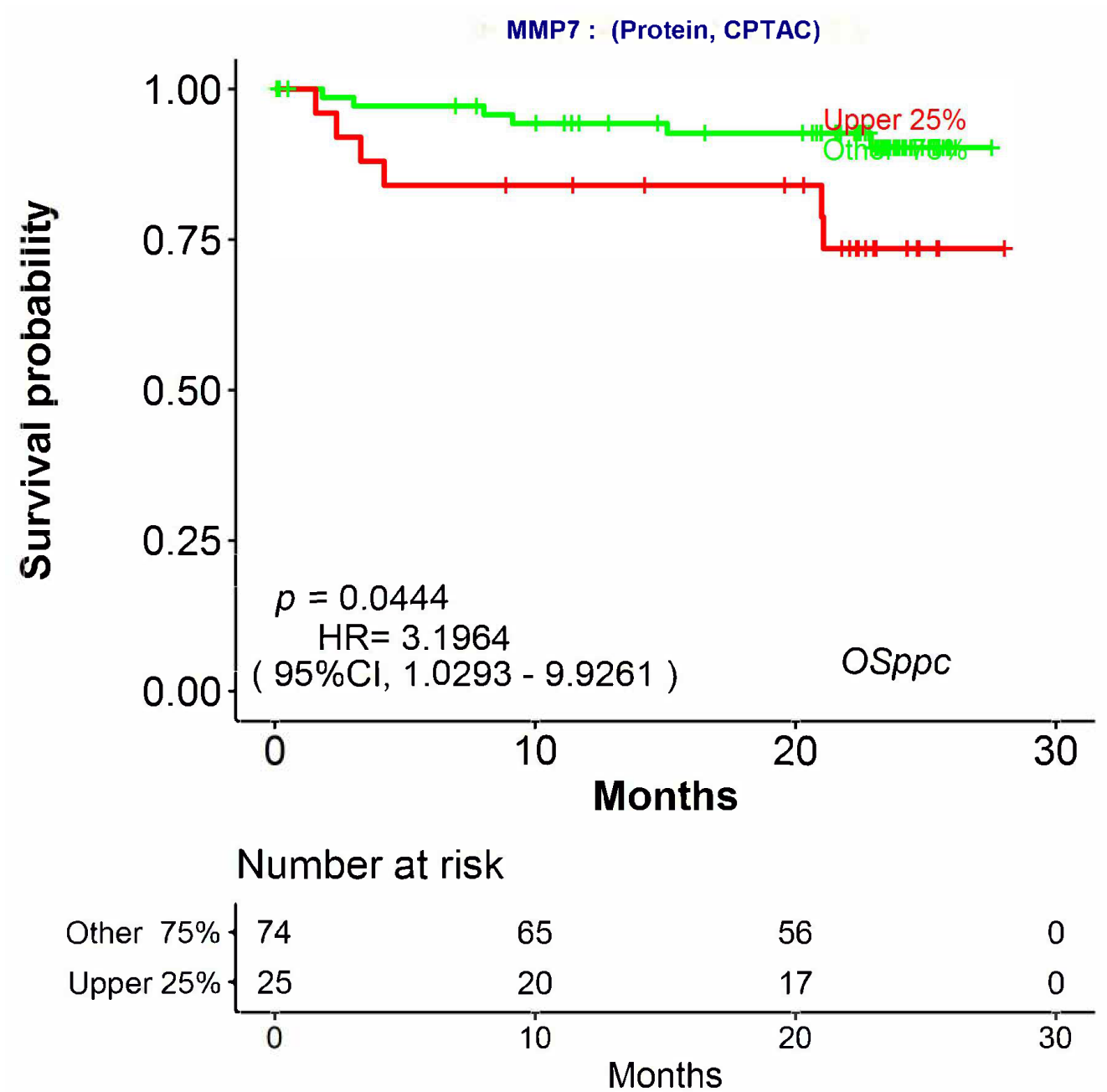

Supplement: Supplementary Figure 2 — Evaluation of the prognostic role of MMP7 in kidney renal clear cell carcinoma (KIRC) at the protein level based on the online platform “OSppc” (https://bioinfo.henu.edu.cn/Protein/OSppc.html). Low protein expression of MMP7 correlated with better overall survival in KIRC (P = 0.0444). [file DataSheet_3.pdf]

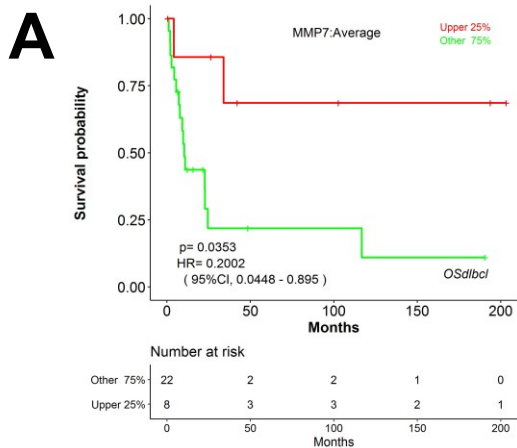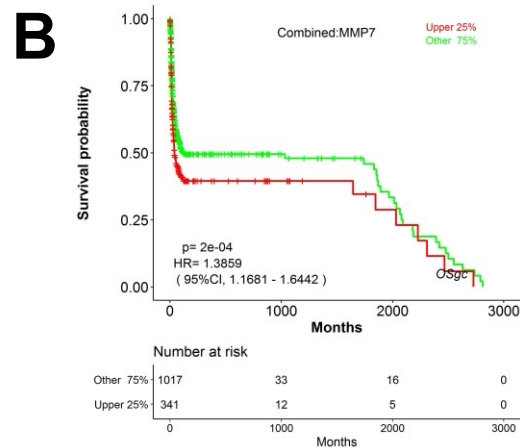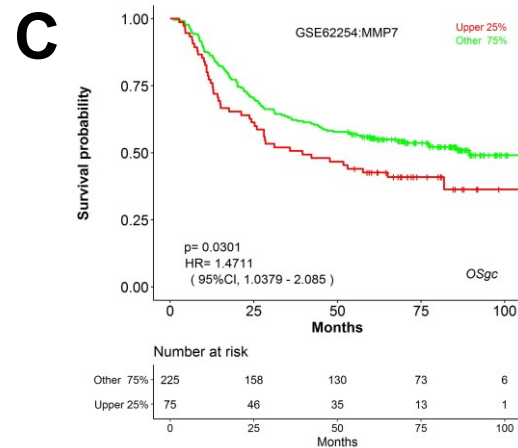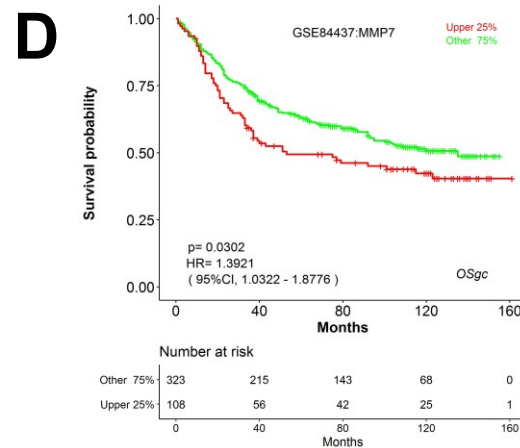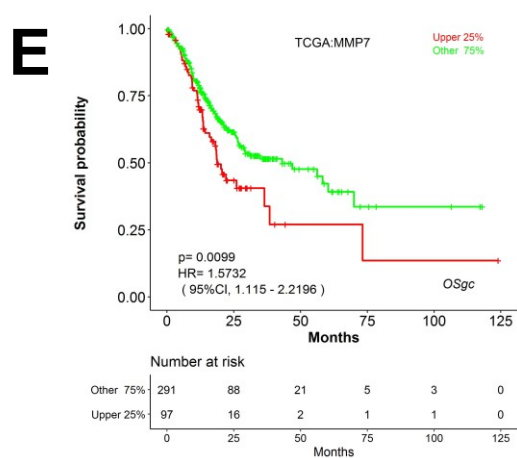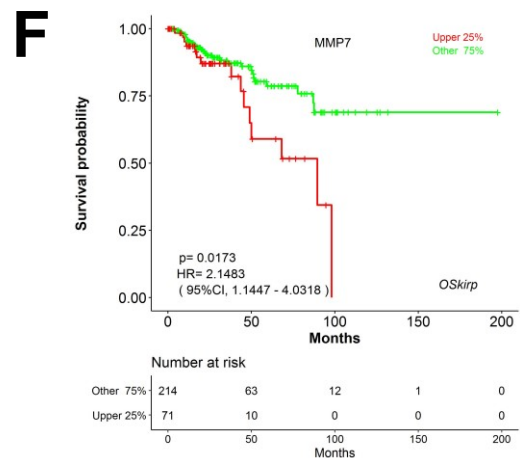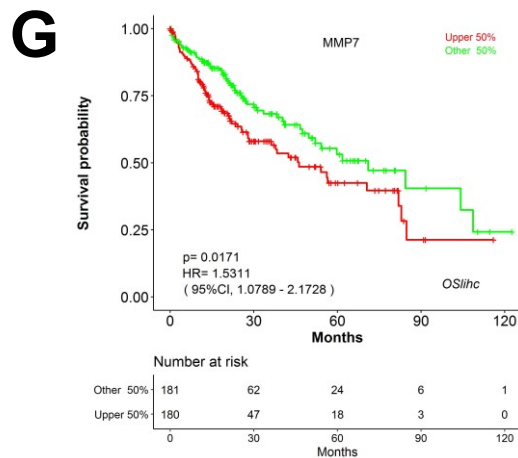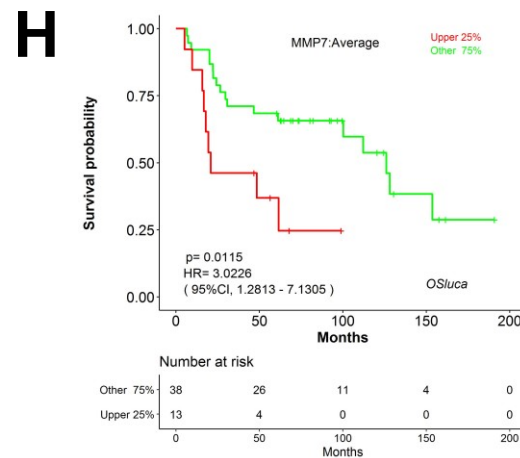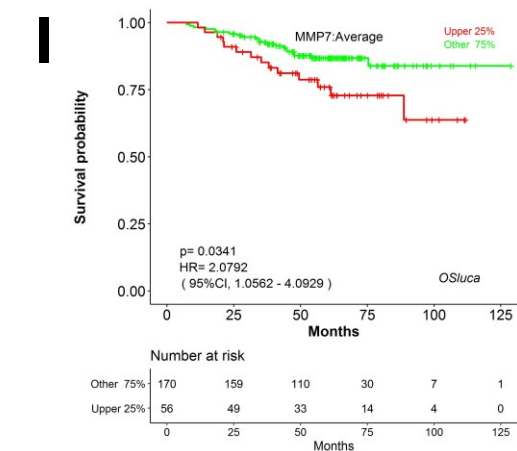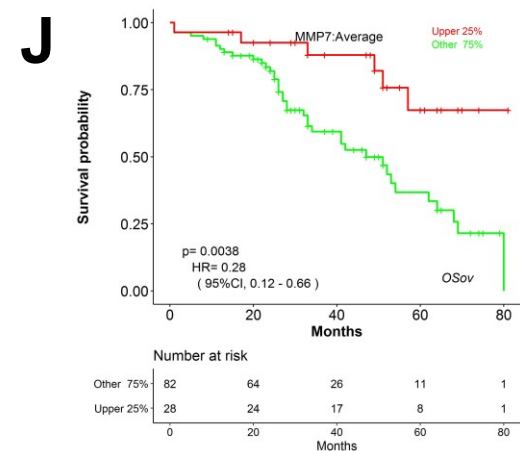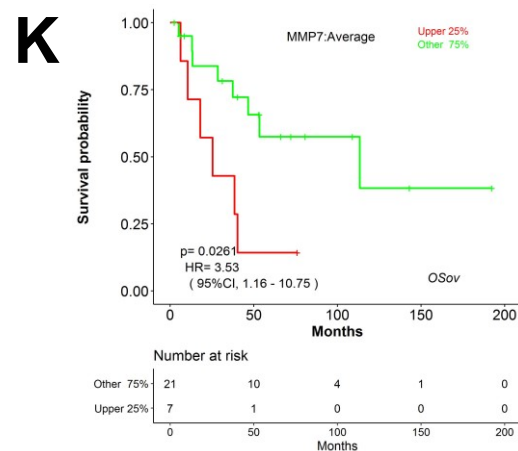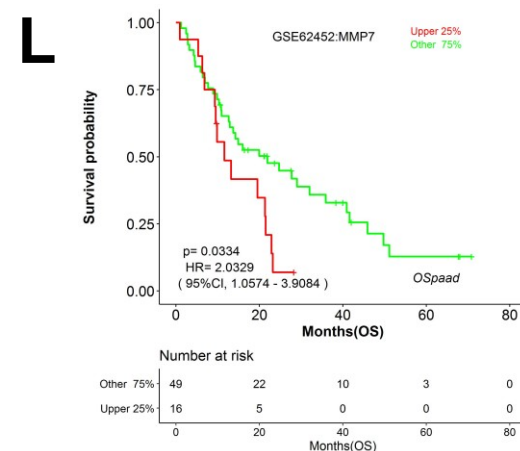

Supplement: Supplementary Figure 3 — Survival analysis of MMP7 in different malignancies based on the web sever of LOGpc with multiple data sources. Higher expression of MMP7 indicated better overall survival in DLBC (A, GSE57611) and OV (J, GSE17260). Poor overall survival correlated with high expression of MMP7 in gastric cancer (B, combined data sources), gastric cancer (C, GSE62254), gastric cancer (D, GSE84437), gastric cancer (E, TCGA), KIRP (F, TCGA), LIHC (G, TCGA), LUCA (H, GSE5123), LUCA (I, GSE31210), OV (K, GSE23554) and PAAD (K, GSE62452). [file DataSheet_4.pdf]
