# Supplementary material for: A Comprehensive Pan-Cancer Analysis of the Tumorigenic Role of Matrix Metallopeptidase 7 (MMP7) Across Human Cancers
Source: Front Oncol. 2022 Jun 17;12:916907. doi: 10.3389/fonc.2022.916907 (PMC9248742; doi:10.3389/fonc.2022.916907)
Supplement: Supplementary file 5 [file Table_1.docx]

**Supplementary Table 1. Clinicopathological information and MMP7 protein expression in STAD and COAD patients.**

| Cancer type | Gender | Age (years) | Operation | Differentiation | Stage | MM7 expression |
| --- | --- | --- | --- | --- | --- | --- |
| STAD | Male | 71 | Laproscopic | Poor-moderate | Ⅲ | High |
| STAD | Male | 58 | Open | Moderate | Ⅰ | High |
| STAD | Male | 56 | Laproscopic | Poor | Ⅲ | High |
| STAD | Female | 67 | Open | Poor-moderate | Ⅲ | High |
| STAD | Male | 72 | Open | Poor-moderate | Ⅲ | High |
| STAD | Female | 61 | Laproscopic | Poor | Ⅲ | High |
| STAD | Female | 45 | Laproscopic | Poor-moderate | Ⅱ | High |
| STAD | Female | 72 | Laproscopic | Poor-moderate | Ⅰ | High |
| STAD | Female | 53 | Laproscopic | Poor-moderate | Ⅰ | High |
| COAD | Male | 66 | Laproscopic | Moderate | Ⅰ | High |
| COAD | Female | 45 | Laproscopic | Poor-moderate | Ⅲ | High |
| COAD | Male | 73 | Laproscopic | Moderate | Ⅲ | High |
| COAD | Female | 70 | Laproscopic | Moderate | Ⅰ | High |
| COAD | Male | 65 | Laproscopic | Moderate | Ⅱ | High |
| COAD | Male | 72 | Laproscopic | Moderate | Ⅳ | High |
| COAD | Male | 66 | Laproscopic | Moderate | Ⅱ | High |
| COAD | Female | 57 | Laproscopic | Moderate | Ⅲ | High |
| COAD | Male | 75 | Laproscopic | Moderate | Ⅱ | High |
