# Supplementary material for: A Comprehensive Pan-Cancer Analysis of the Tumorigenic Role of Matrix Metallopeptidase 7 (MMP7) Across Human Cancers
Source: Front Oncol. 2022 Jun 17;12:916907. doi: 10.3389/fonc.2022.916907 (PMC9248742; doi:10.3389/fonc.2022.916907)
Supplement: Supplementary file 6 [file Table_2.docx]

**Supplementary Table 2. Gene expression, protein level and the roles of MMP7 across all the TCGA cancer types.**

| Cancer types | *MMP7* gene expression | MMP7 protein expression | Roles |
| --- | --- | --- | --- |
| ACC | NS | NA | NS |
| BLCA | NS | NA | NS |
| BRCA | Down-regulated | Down-regulated | NS |
| CESC | NS | NA | NS |
| CHOL | Up-regulated | NA | NS |
| COAD | Up-regulated | Up-regulated | NS |
| DLBC | Up-regulated | NA | Tumor suppressor |
| ESCA | Up-regulated | NA | NS |
| GBM | Up-regulated | NS | NS |
| HNSC | Up-regulated | NA | NS |
| KICH | Down-regulated | NA | NS |
| KIRC | Down-regulated | Down-regulated | Oncogenic |
| KIRP | Up-regulated | NA | Oncogenic |
| LAML | NS | NA | Oncogenic |
| LGG | NS | A | Oncogenic |
| LIHC | Down-regulated | NA | Oncogenic |
| LUAD | Up-regulated | Up-regulated | Oncogenic |
| LUSC | Up-regulated | NA | Oncogenic |
| MESO | NS | NA | NS |
| OV | Up-regulated | Down-regulated | Tumor suppressor |
| PAAD | NS | NA | Oncogenic |
| PCPG | NS | NA | NS |
| PRAD | NS | NA | NS |
| READ | Up-regulated | NA | NS |
| SARC | NS | NA | Oncogenic |
| SKCM | Down-regulated | NA | NS |
| STAD | Up-regulated | NA | Oncogenic |
| TGCT | NS | NA | NS |
| THCA | Up-regulated | NA | NS |
| THYM | NS | NA | Oncogenic |
| UCEC | NS | Up-regulated | NS |
| UCS | NS | NA | NS |
| UVM | NS | NA | NS |

NS, no significance. NA, not available.
